# Phylogenetic Association and Genetic Factors in Cold Stress Tolerance in Campylobacter jejuni

**DOI:** 10.1128/spectrum.02681-22

**Published:** 2022-10-31

**Authors:** Jeong In Hur, Jinshil Kim, Sangryeol Ryu, Byeonghwa Jeon

**Affiliations:** a Department of Food and Animal Biotechnology, Research Institute of Agriculture and Life Sciences, Seoul National University, Seoul, Republic of Korea; b Department of Agricultural Biotechnology, Seoul National University, Seoul, Republic of Korea; c Center for Food and Bioconvergence, Seoul National University, Seoul, Republic of Korea; d Division of Environmental Health Sciences, School of Public Health, University of Minnesota, Minneapolis, Minnesota, USA; University of Guelph

**Keywords:** *Campylobacter jejuni*, cold stress tolerance, ferric enterobactin receptor, CfrA

## Abstract

Campylobacter jejuni is a major foodborne pathogen transmitted to humans primarily via contaminated poultry meat. Since poultry meat is generally processed, distributed, and stored in the cold chain, the survival of C. jejuni at refrigeration temperatures crucially affects human exposure to C. jejuni. Here, we investigated genetic factors associated with cold stress tolerance in C. jejuni. Seventy-nine C. jejuni strains isolated from retail raw chicken exhibited different survival levels at 4°C for 21 days. Multilocus sequence typing (MLST) clonal complex 21 (CC-21) and CC-443 were dominant among cold stress-tolerant strains, whereas CC-45 was common among cold stress-sensitive strains. Genome-wide average nucleotide identity (ANI) analysis identified a phylogenetic cluster associated with cold stress tolerance. Moreover, a pangenome analysis revealed 58 genes distinctively present in the cold stress-tolerant phylogenetic cluster. Among these 58 genes, *cfrA*, encoding the ferric enterobactin receptor involved in ion transport and metabolism, was selected for further analysis. Remarkably, the viability of a Δ*cfrA* mutant at 4°C was significantly decreased, while the levels of total reactive oxygen species and intracellular iron exceeded those of the wild type. Additionally, a knockout mutation of *cfrA* also significantly decreased the viability of three cold stress-tolerant isolates at 4°C, confirming the role of *cfrA* in cold stress tolerance. The results of this study demonstrate that unique phylogenetic clusters of C. jejuni associated with cold stress tolerance exist and that *cfrA* is a genetic factor contributing to cold stress tolerance in C. jejuni.

**IMPORTANCE** The tolerance of foodborne pathogens to environmental stresses significantly affects food safety. Several studies have demonstrated that C. jejuni survives extended exposures to low temperatures, but the mechanisms of cold stress tolerance are not fully understood. Here, we demonstrate that C. jejuni strains in certain phylogenetic groups exhibit increased tolerance to cold stress. Notably, *cfrA* is present in the phylogenetic cluster associated with cold stress tolerance and plays a role in the survival of C. jejuni at low temperatures by alleviating oxidative stress. This is the first study to discover phylogenetic associations involving cold stress tolerance and to identify genetic elements conferring cold stress tolerance to C. jejuni.

## INTRODUCTION

Campylobacter jejuni is a major cause of acute gastroenteritis in humans (1–3). Human infection by C. jejuni is frequently associated with the consumption of contaminated poultry meat (4, 5), manifesting clinical symptoms such as diarrhea, abdominal cramps, and fever (6). In some cases, C. jejuni infection can result in Guillain-Barré syndrome, a neuropathy causing muscular paralysis, as a postinfection complication (7, 8). The food industries in most developed countries have adopted cold-chain processing and distribution of meat products to ensure food safety and quality (3, 9). Although C. jejuni, as a thermotolerant species, can optimally grow at elevated temperatures such as 42°C, the survival of C. jejuni on poultry meat in the cold chain poses a food safety threat (10, 11).

Most foodborne pathogens, such as *Bacillus*, Salmonella, and Escherichia coli, produce cold shock proteins (12–14). When exposed to cold shock, E. coli increases the expression of cold shock proteins such as CspA (15, 16), which helps bacteria survive at low temperatures by disaggregating and reactivating proteins unfolded or misfolded by the temperature downshift (17, 18). The cold stress response is a complicated process involving various genetic elements and gene expression regulation (19, 20). In order to adapt to cold stress environments, for example, Salmonella undergoes extensive gene expression changes involving regulators such as Fur, RpoE, and CsrA (21, 22). As noted above, most human campylobacteriosis cases are caused primarily by the consumption of contaminated poultry. This suggests that despite the lack of cold shock proteins, C. jejuni can successfully survive extended exposures to low temperatures of the cold chain during the distribution and storage of poultry products (11). Studies thus far have shown that oxidative stress defense is associated with cold stress tolerance in Campylobacter (23, 24). Exposure to cold stress increases the expression of oxidative stress defense genes in C. jejuni (23). Moreover, a knockout mutation of *sodB*, encoding superoxide dismutase (SodB), compromises viability after freeze-thaw stress (24).

Iron is essential for various physiological processes; however, excessive iron disrupts redox homeostasis and catalyzes the generation of reactive oxygen species (ROS) via the Fenton reaction under stress conditions (25–27). ROS cause oxidative damage to cellular components such as DNA and proteins and can lead to cell death (28). Since the iron-catalyzed Fenton reaction converts hydrogen peroxide to hydroxyl radicals, the most noxious ROS causing cellular damage, intracellular free iron levels can be correlated with oxidative stress (27). The expression of iron-related genes is elevated in C. jejuni during cold shock (23), suggesting that iron may play an essential role in the adaptation of C. jejuni to cold shock. However, little is understood about how C. jejuni can tolerate low temperatures of the cold chain during foodborne transmission to humans via refrigerated poultry meat.

To fill this knowledge gap, in this study, we investigated cold stress tolerance in 79 C. jejuni strains isolated from retail raw chicken in our previous study (29) and discovered that some strains of C. jejuni are highly tolerant to cold stress. Moreover, cold stress tolerance is associated with specific clonal complexes (CCs), which indicates that strains with cold stress tolerance are phylogenetically related. By comparing 79 C. jejuni isolates and testing them with gene knockout mutations, we show that *cfrA* contributes to cold stress tolerance in C. jejuni. Notably, we demonstrate that intracellular iron and oxidative stress defenses are related to cold stress tolerance driven by *cfrA* in C. jejuni.

## RESULTS

### Phylogenetic association with cold stress tolerance in C. jejuni.

**FIG 1:**
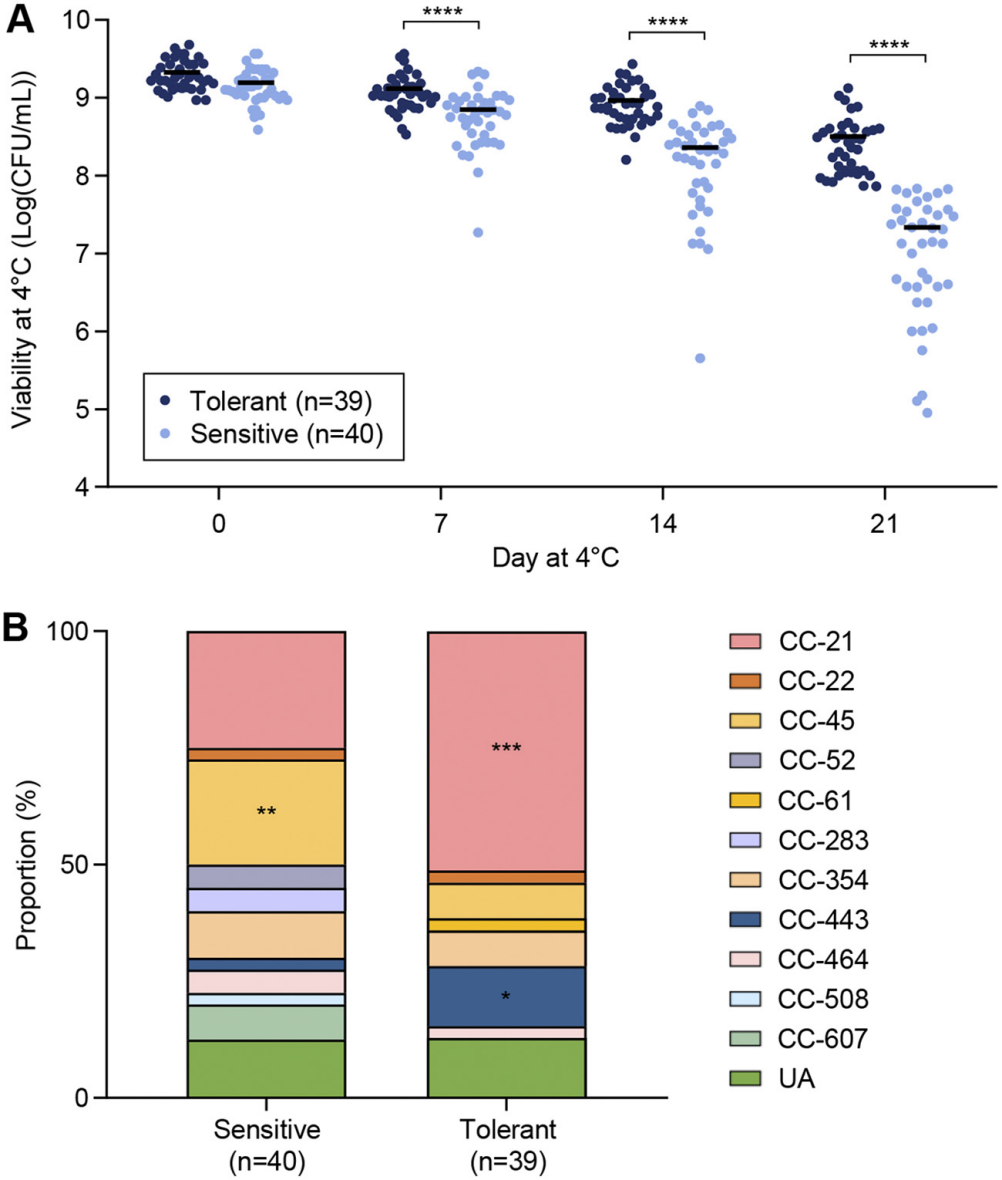
Different levels of cold stress tolerance in C. jejuni isolates from retail chicken and differences in the proportions of MLST clonal complexes (CCs) between cold stress-sensitive and cold stress-tolerant strains. (A) Viable counts of 79 C. jejuni strains were measured at 0, 7, 14, and 21 days of exposure at 4°C. Student’s *t* test was performed to compare the viabilities of cold stress-sensitive and cold stress-tolerant strains. Solid black bars indicate the means. Representative data from three independent experiments with similar results are shown. (B) MLST CCs of 79 C. jejuni strains were compared between cold stress-sensitive and cold stress-tolerant strains. Cold stress-sensitive and cold stress-tolerant strains were grouped based on viability after cold stress exposure for 21 days. A chi-square test was conducted to determine whether the CC proportions were statistically related to cold stress tolerance. *, *P < *0.05; **, *P < *0.01; ***, *P < *0.001; ****, *P < *0.0001. UA, unassigned to any defined CC.

C. jejuni can be isolated from refrigerated poultry meat and various environmental samples from poultry farms, although it is a thermotolerant species (30, 31). Thus, we hypothesized that C. jejuni strains circulating in poultry production may have the ability to tolerate cold temperatures. Using 79 C. jejuni strains isolated from retail raw chicken in our previous study (29), we first measured the survival of C. jejuni at refrigeration temperature for 21 days. Since the tested strains displayed a wide range of viabilities at 4°C, we divided the 79 strains into two groups of equal sizes by their viability at 21 days and designated them cold stress tolerant (*n* = 39) and cold stress sensitive (*n* = 40). The dividing point was approximately 7.0 × 10^7^ CFU/mL on day 21. The viable counts of the cold stress-tolerant strains at 4°C at all sampling times (7, 14, and 21 days) were significantly different from those of the cold stress-sensitive strains (Fig. 1A).

To determine whether cold stress tolerance is related to bacterial phylogeny in C. jejuni, we compared multilocus sequence typing (MLST) CCs between cold stress-tolerant and cold stress-sensitive strains. Notably, CC-21 and CC-443 were predominant in cold stress-tolerant strains (51.3% and 12.8%, respectively), whereas CC-45 was dominant in cold stress-sensitive strains (22.5%) (Fig. 1B). The associations of CC-21, CC-45, and CC-443 with cold stress tolerance were statistically significant (see Fig. S1 in the supplemental material). CC-21 was dominant in both cold stress-tolerant and cold stress-sensitive strains (51.3% and 25.0%, respectively); however, statistical analysis showed that the proportion of CC-21 is significant only in cold stress-tolerant strains (Fig. 1B). These findings demonstrate that some C. jejuni strains are highly tolerant to low temperatures and that cold stress tolerance is phylogenetically associated in C. jejuni.

### C. jejuni strains tolerant or sensitive to cold stress are phylogenetically distinct.

**FIG 2:**
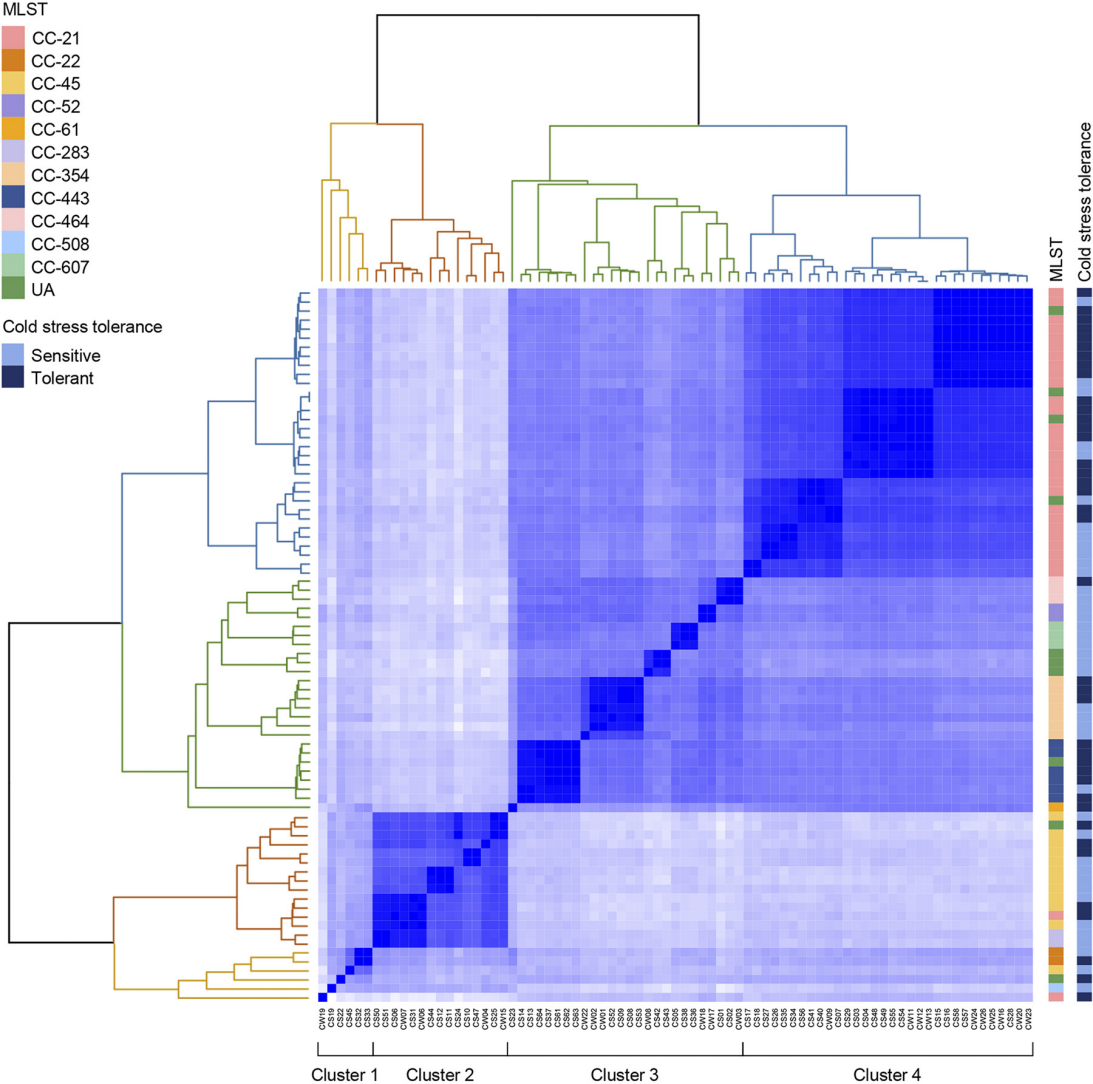
Identification of phylogenetic clusters related to cold stress tolerance in C. jejuni. Genome-wide ANI values separated 79 C. jejuni strains into four clusters: cluster 1 (*n* = 6) (yellow), cluster 2 (*n* = 15) (orange), cluster 3 (*n* = 26) (green), and cluster 4 (*n* = 32) (blue). The information about MLST and cold stress tolerance for 79 C. jejuni strains is indicated on the right side of the heat map. CC, clonal complex; UA, unassigned to any defined clonal complex.

**FIG 3:**
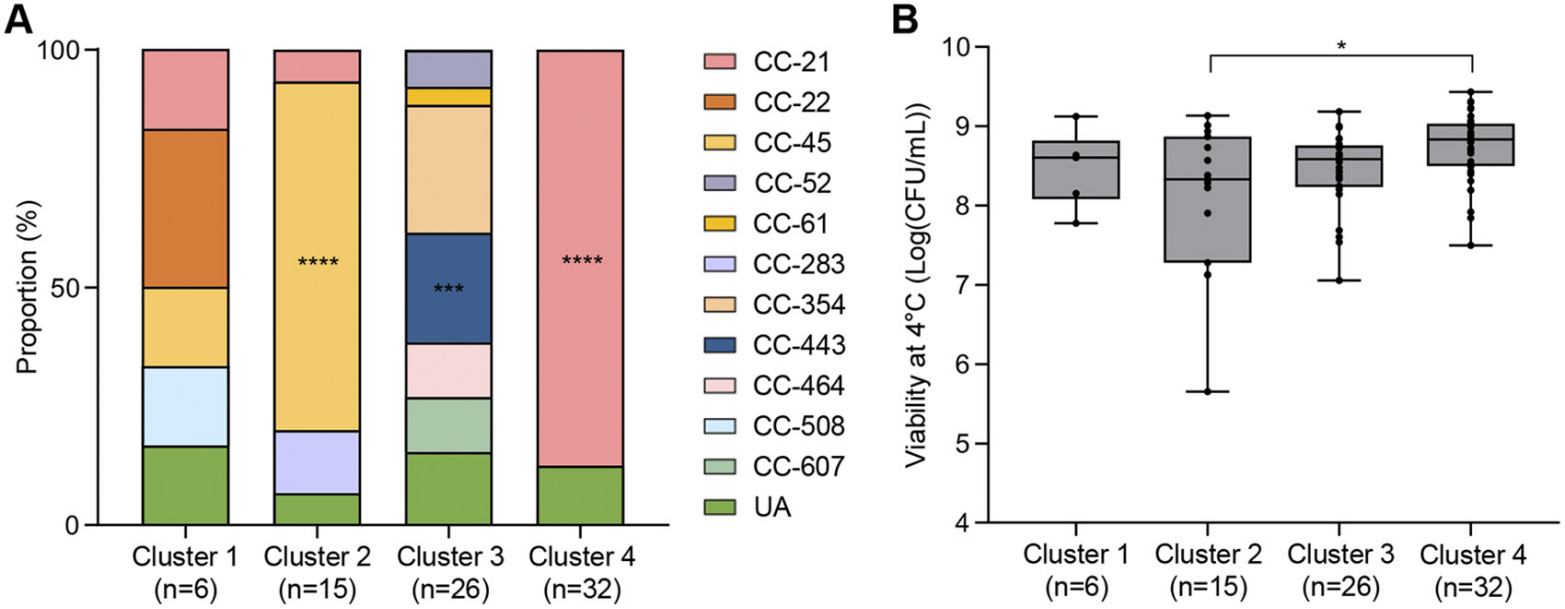
Association of phylogenetic clusters with MLST clonal complexes (CCs) and cold stress tolerance. (A) Proportions of MLST CCs in the four phylogenetic clusters. A chi-square test was conducted for statistical analysis. (B) Viability at 4°C for 21 days of C. jejuni strains of the four phylogenetic clusters. The results indicate means and standard deviations. Student’s *t* test was performed to compare viabilities between two clusters. *, *P < *0.05; ***, *P < *0.001; ****, *P < *0.0001. UA, unassigned to any defined CC.

We performed genome-wide average nucleotide identity (ANI) analysis to further investigate the phylogenetic association with cold stress tolerance. As a result, we identified four phylogenetic clusters that are distinctly separate below the 98% ANI threshold: cluster 1 (*n* = 6), cluster 2 (*n* = 15), cluster 3 (*n* = 26), and cluster 4 (*n* = 32) (Fig. 2). Interestingly, the phylogenetic clusters were related to MLST CCs and cold stress tolerance. When MLST CCs were compared, cluster 2 showed a significantly high proportion of CC-45, while CC-443 and CC-21 were highly prevalent in cluster 3 and cluster 4, respectively (Fig. 3A). Consistent with the patterns of cold stress tolerance of these CCs (Fig. 1B), cluster 2 and cluster 4 consisted mostly of cold stress-sensitive and cold stress-tolerant strains, respectively (Fig. S2). Moreover, the viabilities of C. jejuni after 21 days of exposure to cold stress were significantly different between cluster 2 and cluster 4 (Fig. 3B). These results suggest that cold stress tolerance is associated with genetic backgrounds in C. jejuni.

### Genetic elements are unique to cold stress-tolerant strains of C. jejuni.

**FIG 4:**
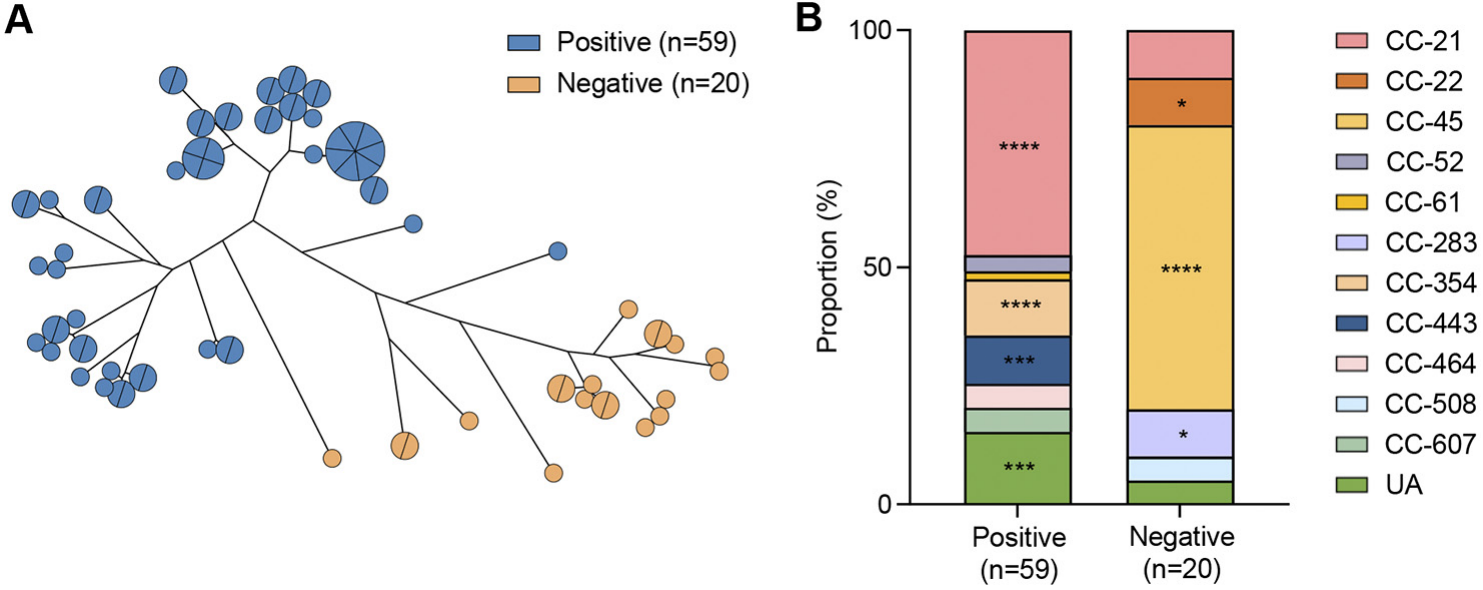
Phylogenetic distinction and MLST clonal complex (CC) composition depending on the presence of *cfrA*. (A) A minimum-spanning tree was generated using the core-gene alignment obtained from the pangenome analysis. (B) MLST CCs of 79 C. jejuni strains were compared between *cfrA*-positive and *cfrA*-negative phylogenies. A chi-square test was conducted for comparisons of the proportions of the CCs. *, *P < *0.05; ***, *P < *0.001; ****, *P < *0.0001. UA, unassigned to any defined CC.

Cluster 2 and cluster 4 were phylogenetically distant (Fig. 2) and showed significantly different levels of cold stress tolerance (Fig. 3B). Thus, we conducted a pangenome analysis to identify genes potentially associated with cold stress tolerance by comparing the two clusters. This analysis revealed 58 genes that are present in the cold-tolerant cluster (i.e., cluster 4) and absent from the cold-sensitive cluster (i.e., cluster 2) (Table 1; Fig. S3). These 58 genes are involved in various functions, including inorganic ion transport and metabolism, amino acid transport and metabolism, defense mechanisms, transcription, and carbohydrate transport and metabolism (Table 1). Previous studies have shown that oxidative stress responses affect the survival of Campylobacter under cold stress conditions (24, 32), and iron metabolism is closely related to oxidative stress defense (25–27). Therefore, among the 58 genes, we decided to focus on the genes related to iron metabolism. Specifically, a previous study showed that cold shock significantly increases the level of *cfrA* transcription in C. jejuni (23). Furthermore, *cfrA* is essential for bacterial survival under stress conditions during host colonization (33). Based on these studies, we selected *cfrA*, encoding a ferric enterobactin receptor, for further investigation. When we examined the occurrence of *cfrA* in the 79 C. jejuni isolates, there was a clear separation of phylogenetic groups (Fig. 4A). The strains lacking *cfrA* belonged predominantly to CC-45 (60.0%), the CC associated with cold stress sensitivity, and the strains harboring *cfrA* belonged predominantly to CC-21 (47.5%), UA (unassigned) (15.3%), CC-354 (11.9%), and CC-443 (10.2%) (Fig. 4B). Notably, these results are consistent with the results of viability assays and the ANI analysis, which also show that strains in CC-45 are related to cold stress sensitivity and that those in CC-21 and CC-443 tend to be tolerant to cold stress (Fig. 1 and 2). These data suggest that *cfrA* can be involved in cold stress tolerance in C. jejuni.

**TABLE 1:** Fifty-eight genes present in the cold-tolerant cluster and absent in the cold-sensitive cluster

Function(s) and locus tag	Gene	Description of the gene product^*a*^
Inorganic ion transport and metabolism		
*cj0444*	Pseudogene	TonB-dependent receptor
*cj0676*	*kdpA*	Potassium-transporting ATPase subunit KdpA
*cj0755*	*cfrA*	Ferric enterobactin receptor CfrA
*cj1040c*		MFS transporter
*cj1415c*	*cysC*	Adenylyl-sulfate kinase
Amino acid transport and metabolism		
*cj0029*	*ansA*	Type II asparaginase
*cj0481*	*dapA*	Dihydrodipicolinate synthase family protein
*cj0763c*	*hisS*	Histidine-tRNA ligase
*cj0817*	*glnH*	Transporter substrate-binding domain-containing protein
*cj1726c*	*metA*	Homoserine *O*-succinyltransferase
*cj1727c*	*metB*	*O*-Acetylhomoserine aminocarboxypropyltransferase/cysteine synthase
Defense mechanisms		
*cj0139*		Hypothetical protein
*cj0690c*		SAM-dependent DNA methyltransferase
*cj1549c*	*hsdR*	Type I restriction endonuclease subunit R
*cj1551c*	*hsdS*	Restriction endonuclease subunit S
*cj1553c*	*hsdM*	SAM-dependent DNA methyltransferase
Transcription		
*cj0480c*		IclR family transcriptional regulator
*cj0757*	*hrcA*	HrcA family transcriptional regulator
*cj1552c*	*mloB*	Transcriptional regulator
*cj1556*		Helix-turn-helix transcriptional regulator
Carbohydrate transport and metabolism		
*cj0482*	*uxaA*′	UxaA family hydrolase
*cj0483*	*uxaA*′	UxaA family hydrolase
*cj0484*		MFS transporter
Secondary metabolite biosynthesis, transport, and catabolism		
*cj0170*		Methyltransferase domain-containing protein
*cj1325*		Methyltransferase domain-containing protein
*cj1420c*		Class I SAM-dependent methyltransferase
Energy production and conversion		
*cj0490*	*ald*′	Aldehyde dehydrogenase
*cj1585c*		FAD-binding oxidoreductase
Nucleotide transport and metabolism		
*cj0766*	Pseudogene	Putative arylsulfate sulfotransferase
*cj0381c*	*pyrF*	Orotidine-5′-phosphate decarboxylase
General function prediction only		
*cj0054c*		TIGR00730 family Rossman fold protein
*cj1555c*		NAD(P)-dependent oxidoreductase
Lipid transport and metabolism		
*cj0485*		SDR family oxidoreductase
Posttranslational modification, protein turnover, and chaperones		
*cj1725*		NAD(P)/FAD-dependent oxidoreductase
Intracellular trafficking, secretion, and vesicular transport		
*cj0969*	Pseudogene	Hemagglutination domain protein
Unknown function		
*cj0055c*		Hypothetical protein
*cj0056c*		Hypothetical protein
*cj0247c*		Chemotaxis protein
*cj0380c*		Hypothetical protein
*cj0425*		Hypothetical protein
*cj0565*		Hypothetical protein
*cj0566*		Hypothetical protein
*cj0568*		Hypothetical protein
*cj0569*		Hypothetical protein
*cj0617*		DUF2920 family protein
*cj0685c*	*cipA*	DUF2972 domain-containing protein
*cj0740*		Hypothetical protein
*cj0818*		Hypothetical protein
*cj0859c*		Hypothetical protein
*cj0866*	Pseudogene	Arylsulfate sulfotransferase
*cj0970*		Hypothetical protein
*cj0972*		Hypothetical protein
*cj0978c*		Putative lipoprotein
*cj1122c*		Hypothetical protein
*cj1456c*		Hypothetical protein
*cj1550c*	*rloH*	AAA family ATPase
*cj1558*		Permease
*cj1714*		Small hydrophobic protein

a MFS, major facilitator superfamily; SAM, *S*-adenosylmethionine; FAD, flavin adenine dinucleotide; SDR, short-chain dehydrogenases/reductases.

### CfrA contributes to the survival of C. jejuni at refrigeration temperatures.

**FIG 5:**
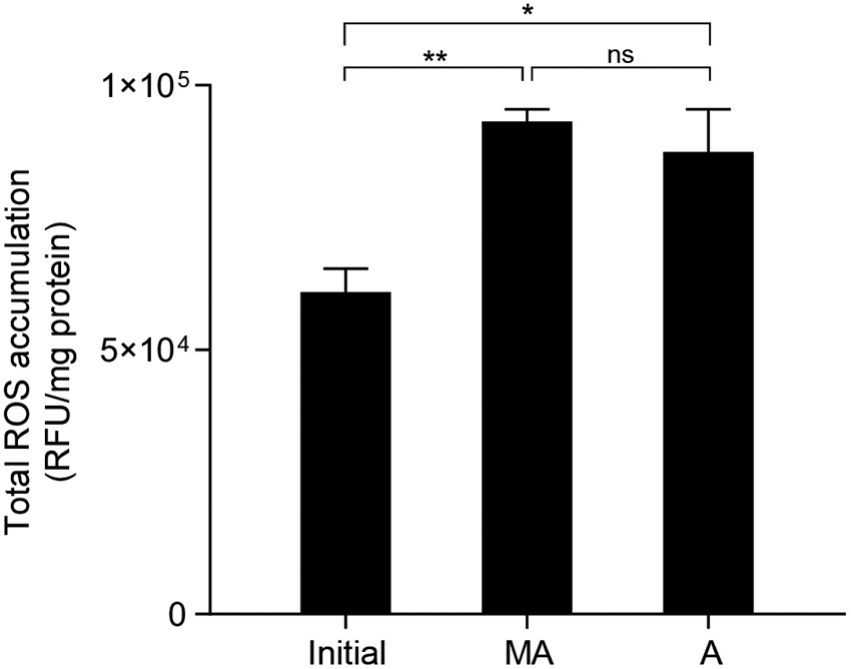
Increased oxidative stress after exposure to cold stress in C. jejuni under microaerobic and aerobic conditions. Total ROS accumulation levels were measured before (Initial) and after exposure to cold stress for 4 days under microaerobic (MA) or aerobic (A) conditions. The experiment was repeated three times. Each bar indicates the standard error of the mean. Student’s *t* test was performed for statistical analysis. *, *P < *0.05; **, *P < *0.01; ns, nonsignificant. RFU, relative fluorescence units.

Previous studies showed that a *sodB* mutation compromises the survival of C. jejuni at refrigeration temperatures, indicating that oxidative stress defense is related to cold stress tolerance (18, 23). Intracellular iron affects oxidative stress through Fenton chemistry (34, 35). Therefore, we hypothesized that *cfrA* may be involved in cold stress tolerance by affecting the oxidative stress response in C. jejuni at refrigeration temperatures. Before testing this hypothesis using the Δ*cfrA* mutant, we questioned whether cold stress could induce oxidative stress in C. jejuni. We observed that exposure to cold stress at 4°C led to ROS accumulation in C. jejuni (Fig. 5), suggesting that the ability of C. jejuni to detoxify ROS is reduced at low temperatures. The levels of total ROS accumulation at 4°C were similar under microaerobic and aerobic conditions (Fig. 5). These results indicate that refrigeration leads to ROS accumulation in C. jejuni regardless of the oxygen level.

**FIG 6:**
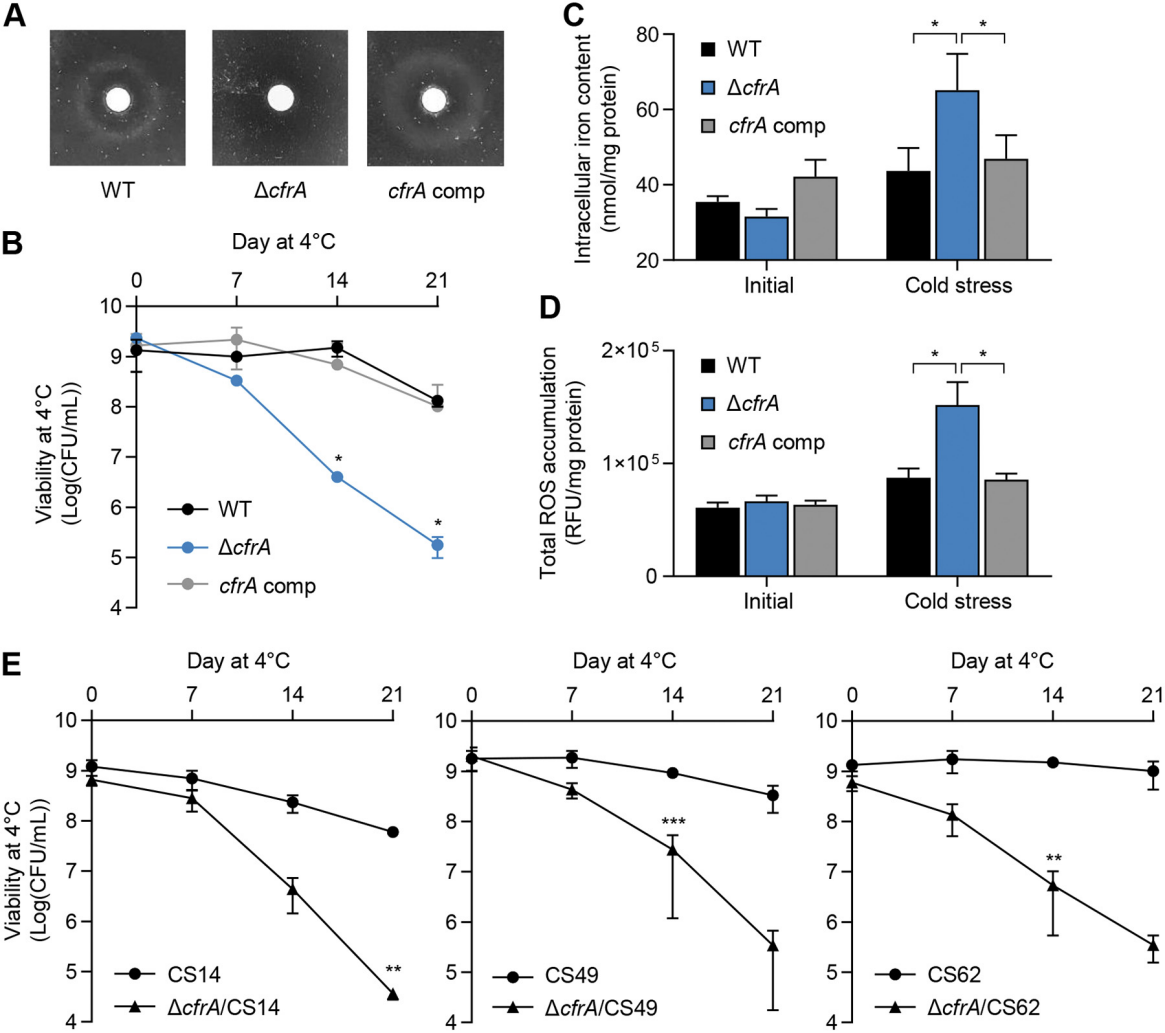
Contribution of *cfrA* to cold stress tolerance in C. jejuni. (A) Inability of a Δ*cfrA* mutant to take up enterobactin. (B) Defective cold stress tolerance in the Δ*cfrA* mutant. The asterisks indicate a significant difference in viability between the Δ*cfrA* mutant and the WT at the same sampling time. (C) Intracellular iron levels in C. jejuni before and after exposure to cold stress at 4°C for 4 days. (D) Total ROS accumulation in C. jejuni before and after exposure to cold stress for 4 days. (E) Significant defects in cold stress tolerance in three cold stress-tolerant strains of C. jejuni. The asterisks indicate the statistical significance of differences in viability between the Δ*cfrA* mutant and the wild type at the same sampling time after exposure to cold stress. The experiment was repeated three times and produced similar results. The error bars show the standard errors of the means. Student’s *t* test was performed for statistical analysis. *, *P < *0.05; **, *P < *0.01; ***, *P < *0.001. WT, C. jejuni NCTC 11168 wild type; Δ*cfrA*, Δ*cfrA* mutant; *cfrA* comp, *cfrA*-complemented strain.

To further examine whether *cfrA* is involved in cold stress tolerance, we constructed a Δ*cfrA* mutant. In addition to the genetic confirmation of a mutation by sequencing (data not shown), the mutation was confirmed phenotypically by observing a defect in the uptake of the ferric enterobactin complex in the Δ*cfrA* mutant (Fig. 6A). Remarkably, the viability of the Δ*cfrA* mutant at 4°C was significantly decreased compared to that of the wild type (WT) (36) (Fig. 6B). The genetic complementation of the Δ*cfrA* mutant with an intact copy of *cfrA* fully restored cold stress tolerance to the WT level (Fig. 6B). Since *cfrA* is related to iron metabolism, we measured the intracellular iron level before and after exposure to cold stress. Interestingly, the Δ*cfrA* mutation significantly elevated the iron level (Fig. 6C). These results suggest that *cfrA* is associated with the control of intracellular iron levels in C. jejuni under cold stress conditions. Moreover, exposure to cold stress significantly increased the ROS levels in the Δ*cfrA* mutant compared to the WT (Fig. 6D). These results suggest that C. jejuni confronts increased oxidative stress at cold temperatures and that *cfrA* contributes to cold stress tolerance by controlling intracellular iron and oxidative stress.

Finally, we confirmed the role of *cfrA* in cold stress tolerance using three cold stress-tolerant isolates. The three strains were selected from CCs that comprise large proportions of the cold stress-tolerant strains: CS14 (CC-443), CS49 (CC-21), and CS62 (CC-443). We constructed Δ*cfrA* mutants of the three cold stress-tolerant strains to validate the role of *cfrA* in cold stress-tolerant strains. Notably, the knockout mutation of *cfrA* significantly compromised the viability of the three cold stress-tolerant strains of C. jejuni at 4°C compared to their WT strains (Fig. 6E). These data suggest that *cfrA* contributes to cold stress tolerance in C. jejuni by alleviating oxidative stress.

## DISCUSSION

C. jejuni is a major foodborne pathogen transmitted to humans via contaminated poultry meat. Considering the use of the cold chain to process and distribute poultry meat, cold stress is one of the major stress conditions that C. jejuni must overcome during foodborne transmission to humans. However, little attention has been given to cold stress tolerance in C. jejuni. Here, we tested cold stress tolerance in 79 C. jejuni strains isolated from retail raw chicken and discovered that the level of cold stress tolerance varies in C. jejuni depending on the strain (Fig. 1A). Moreover, strains in CC-21 and CC-443 were significantly more likely to show cold stress tolerance, and those in CC-45 were more likely to exhibit cold stress sensitivity (Fig. 1B). These data suggest that genetic elements involved in cold stress tolerance may exist in C. jejuni. A previous study also showed that C. jejuni strains belonging to CC-21 survived better at 4°C than those belonging to CC-45 (37). Phylogenetic studies demonstrated that CC-21 and CC-443 are closely related to each other, whereas CC-45 is more distantly related (38, 39). CC-21 and CC-45 are the major generalist CCs occupying the diverse populations of C. jejuni isolated from multiple different hosts such as chickens, cattle, and wild birds (40–42). CC-443 is frequently associated with chickens (39). An MLST analysis of 1,215 isolates from human campylobacteriosis cases in New Zealand over 9 years showed that CC-45 is characteristic in summer, while CC-21 peaks in late autumn to early winter, exhibiting the seasonal prevalence of C. jejuni strains belonging to CC-21 and CC-45 (43). A similar pattern of summer seasonality of CC-45 has also been reported in the United Kingdom (36). Based on the association of CC-45 with cold stress sensitivity revealed in this study (Fig. 1B), it can be speculated that C. jejuni strains belonging to CC-45 may be less prevalent in poultry production environments in winter and may cause human infections primarily in summer.

The phylogenetic analysis using whole-genome sequences divided the 79 strains into four clusters based on the ANI analysis (Fig. 2) and identified two clusters associated with cold stress tolerance (Fig. 3). A comparison of the genome sequences of the two clusters led to the identification of 58 genes present in C. jejuni strains in the cold stress-tolerant cluster and absent from the strains in the cold stress-sensitive cluster (Table 1). Based on previous efforts to identify genes involved in human campylobacteriosis (44, 45), interestingly, most of the 58 genes unique to the cold stress-tolerant cluster are present only in clinical isolates and absent from nonclinical isolates, including *kpsA* (encoding a potassium-transporting ATPase subunit), *uxaA* (encoding a UxaA family hydrolase), *cfrA* (encoding a ferric enterobactin receptor), and others (46). Although it remains unexplained whether these genes are related to the pathogenicity of C. jejuni, it can be speculated that cold stress-tolerant strains are more likely to cause human infection than cold stress-sensitive strains because cold stress tolerance enables C. jejuni to survive on poultry meat, the primary cause of campylobacteriosis, in the food supply chain, increasing the chances of human exposure to C. jejuni.

Notably, our findings demonstrate that *cfrA* plays a role in cold stress tolerance in C. jejuni. In a previous study, genes related to iron metabolism, including *cfrA*, were found to be crucial for bacterial survival under stressful conditions during host colonization (33). The phylogenetic analysis of the core-gene alignment shows a clear distinction between the *cfrA*-positive and *cfrA*-negative phylogenies (Fig. 4A). CC-21 and CC-443, which are correlated with cold stress tolerance, and CC-45, which is correlated with cold stress sensitivity, are separated based on the presence of *cfrA* (Fig. 4B). Remarkably, the viability of the Δ*cfrA* mutant at 4°C was significantly compromised compared to that of the WT (Fig. 6B). The Δ*cfrA* mutation also reduced cold stress tolerance in cold stress-tolerant isolates (Fig. 6E). Altogether, these results are the first to present the role of CfrA in cold stress tolerance in C. jejuni. Here, it is noteworthy that not all *cfrA*-negative strains are cold stress sensitive, although *cfrA*-negative strains were dominantly (64%) cold stress sensitive (data not shown). This may be because cold stress tolerance is a complicated process (23, 47, 48), and there can be other genetic factors involved in cold stress tolerance.

Previous studies have shown the association of oxidative stress defense with cold stress tolerance in C. jejuni. In particular, a mutation of *sodB*, encoding superoxide dismutase, makes Campylobacter susceptible to freeze-thaw stress (24, 32). We found that oxidative stress is increased when C. jejuni is exposed to refrigeration temperatures (Fig. 6D). The levels of total ROS accumulation were similar under microaerobic and aerobic conditions (Fig. 5), indicating that oxidative stress is increased in C. jejuni at refrigeration temperatures regardless of the oxygen level. A similar observation has been reported for another bacterium, where growth at 4°C increased oxidative stress and generated ROS in Pseudomonas fluorescens MTCC 667, an isolate from Antarctica (49). Presumably, reduced carbon metabolism at low temperatures may decrease the formation of the reducing compounds NADH and reduced flavin adenine dinucleotide (FADH_2_), subsequently increasing oxidative stress. In addition, we also observed that exposure to refrigeration temperatures increased the intracellular level of iron in C. jejuni (Fig. 6C). A knockout mutation of *cfrA* increased the levels of iron and total ROS (Fig. 6C and D), which may decrease viability at refrigeration temperatures because an iron upshift leads to oxidative stress and can trigger cell death. These data suggest that CfrA contributes to the control of intracellular iron and redox homeostasis in C. jejuni at refrigeration temperatures.

In summary, we demonstrated for the first time the phylogenetic association with cold stress tolerance in C. jejuni and showed that specific CCs are associated with cold stress tolerance. We also identified genes unique to the cold stress-tolerant cluster. Finally, we revealed that CfrA contributes to cold stress tolerance by controlling intracellular iron levels and oxidative stress. Future studies will need to elucidate the molecular mechanisms of cold stress tolerance driven by CfrA.

## MATERIALS AND METHODS

### Bacterial strains and culture conditions.

Seventy-nine Campylobacter jejuni strains previously isolated from retail raw chicken (29) were used in this study. C. jejuni NCTC 11168 was used as a reference strain in this study. The C. jejuni strains were routinely grown on Mueller-Hinton (MH) agar (Oxoid, Hampshire, UK) at 42°C for 18 to 24 h under microaerobic conditions (85% N_2_, 5% O_2_, and 10% CO_2_) generated by the Anoxomat system (Mart Microbiology BV, Lichtenvoorde, The Netherlands).

### Cold stress tolerance test of C. jejuni.

The survival of C. jejuni at 4°C was measured as described previously (50), with slight modifications. Briefly, a culture grown overnight on MH agar was resuspended in MH broth to an optical density at 600 nm (OD_600_) of 0.1 (ca. 10^9^ CFU/mL). The bacterial suspension was transferred to multiple 96-well plates in 200 μL aliquots. To prevent sample desiccation, the outer wells were filled with an equal volume of distilled water, and a container with water was placed near the 96-well plates. Wooden sticks were placed under both sides of the lids of the 96-well plates to improve air circulation. The 96-well plates were incubated at 4°C under aerobic conditions, and samples were taken after 0, 7, 14, and 21 days for serial dilution and bacterial counting. Since there were no criteria for cold stress tolerance, we divided the strains into two groups of equal sizes based on viability; the CFU value used to divide the groups was approximately 7.0 × 10^7^ CFU/mL after 21 days. The strains with viable cells at >7.0 × 10^7^ CFU/mL after 21 days were called cold stress-tolerant strains, while those with fewer viable cells than this were called cold stress-sensitive strains.

### Whole-genome sequencing.

Genomic DNA (gDNA) was extracted using a NucleoSpin microbial DNA kit (Macherey-Nagel, PA, USA) and the TissueLyser II system (Qiagen, Hilden, Germany) according to the manufacturers’ instructions. A NanoDrop spectrophotometer (Thermo Fisher Scientific, OH, USA), gel electrophoresis, and a Qubit fluorometer (Thermo Fisher Scientific, OH, USA) were used to evaluate the quality of the gDNA. After quality control of the gDNA, the DNA library was prepared using the TruSeq Nano DNA LT library preparation kit (Illumina, CA, USA) according to the TruSeq Nano DNA library preparation protocol. The quality of the libraries was assessed on a 2100 Bioanalyzer system with a DNA1000 chip (Agilent Technologies, CA, USA). Next, the constructed DNA libraries were sequenced with a 2× 150 bp read length using the NextSeq 500 sequencing system (Illumina, CA, USA).

### Bioinformatics analysis.

Trimming and *de novo* assembly of raw reads generated from whole-genome sequencing were performed using CLC Genomics Workbench v20 with default parameters. Next, the assembled genomes were annotated using Prokka v1.14.6 with default parameters. To specify the degree of overall relatedness among genomes, we estimated the genome-wide ANI using FastANI v1.33. ANI analysis estimates the average nucleotide identity of all orthologous genes shared between any two genomes. Organisms belonging to the same species typically exhibit ≥95% ANI. Pairwise ANI values were visualized using a heat map generated by ComplexHeatmap v2.2.0 and gplots v3.3.5 in R, dividing the strains into four phylogenetic clusters. In a search for characteristic genes present in the cold-tolerant cluster, pangenome analysis was performed using Roary v3.11.2. For comparative analyses of the presence or absence of *cfrA*, minimum-spanning trees were generated and visualized in GrapeTree v1.5.0 with the core-genome alignment obtained from Roary. Only the strains for which the presence or absence of *cfrA* was confirmed by PCR were used for the analysis described above. The primer sets are listed in Table S1 in the supplemental material.

### Construction of Δ*cfrA* mutants and a *cfrA*-complemented strain.

A suicide plasmid carrying *cfrA* was constructed as described previously (51). Briefly, *cfrA* and its flanking region were amplified from C. jejuni by PCR with GXL polymerase (TaKaRa, Tokyo, Japan) using the primers presented in Table S1. After digestion with SalI and BamHI, the PCR products were each ligated to pUC19 that had been treated with the same enzymes. The pUC19 plasmid containing *cfrA* was amplified by PCR from inside the gene with inverse primers using the same polymerase and ligated to a kanamycin cassette from pMW10. The suicide vectors were commercially sequenced by Bionics (Seoul, Republic of Korea). These three plasmids were used as suicide vectors, and each vector was introduced into the WT strain by electroporation. The C. jejuni culture was grown on MH agar plates containing kanamycin (50 μg/mL) to screen for Δ*cfrA* mutants. The *cfrA* mutation was confirmed by PCR and sequencing.

The complementation strain was constructed as previously described (52). Briefly, DNA fragments containing an intact copy of *cfrA* were amplified with primer pairs and cloned into the NotI site on a pUC19 derivative carrying an rRNA gene cluster (53, 54). Plasmids carrying *cfrA* were sequenced by Bionics (Seoul, Republic of Korea) and used as complementation vectors. The complementation vectors were introduced into *cfrA* knockout mutants by electroporation. To screen for *cfrA* complementation strains, the Campylobacter culture was grown on MH agar plates containing kanamycin (50 μg/mL) and chloramphenicol (12.5 μg/mL). The complementation of *cfrA* was confirmed by PCR and sequencing.

### Measurement of ROS levels.

ROS levels were measured as described previously, with slight modifications (55). The total ROS accumulation level was measured using the fluorescent dye CM-H_2_DCFDA (chloromethyl 2′,7′-dichlorodihydrofluorescein diacetate) (Thermo Fisher Scientific, OH, USA). C. jejuni was prepared as a culture grown overnight on MH agar and resuspended in MH broth to an OD_600_ of 0.1. The bacterial suspension was transferred to a disposable culture tube (Kimble, NJ, USA) and incubated at 4°C. Samples were taken before and after exposure to cold stress for 4 days. After treatment with 10 μM CM-H_2_DCFDA dye for 30 min at room temperature, fluorescence was measured using a SpectraMax i3 platform (Molecular Devices, CA, USA) at 495 nm excitation and 527 nm emission wavelengths. The fluorescence levels were normalized to the protein amounts determined using the Bradford assay (Bio-Rad, CA, USA).

### Growth promotion assay.

As previous studies demonstrated that Campylobacter used ferric enterobactin as the sole source of iron during growth promotion assays (56), we measured the growth of C. jejuni strains as previously described (57). Briefly, a culture grown overnight on MH agar was resuspended in MH broth to an OD_600_ of 0.1. C. jejuni cells were grown in a disposable glass tube to log phase. Deferoxamine mesylate salt (DFO) (Sigma-Aldrich, MO, USA), a chelator, was added to melted MH agar at a final concentration of 20 μM. The cells were mixed with DFO-containing MH agar and adjusted to approximately 10^7^ CFU/mL. Each sample mixture was poured into petri dishes for solidification. A sterile disk containing 25 μL of enterobactin (2 mM) (Sigma-Aldrich, MO, USA) was placed on the surface of the agar in each dish. Autoclaved distilled water was used instead of enterobactin as a negative control.

### Measurement of intracellular iron levels.

Levels of intracellular iron were measured as described previously (58). A culture grown overnight on MH agar was resuspended in MH broth to an OD_600_ of 0.1. C. jejuni cells were transferred to disposable culture tubes (Kimble, NJ, USA) in 3 mL aliquots and incubated at 4°C. Samples were taken before and after exposure to cold stress for 4 days. Briefly, the samples were washed twice with ice-cold phosphate-buffered saline (PBS) and disrupted with a sonicator. A standard curve was obtained by dilution with a 1 mM FeCl_3_ (Sigma-Aldrich, MO, USA) standard solution. The samples were mixed with an iron detection reagent (6.5 mM ferrozine, 6.5 mM neocuproine, 2.5 M ammonium acetate, and 1 M ascorbic acid) and incubated at room temperature for 30 min. The absorbance was measured using a SpectraMax i3 platform (Molecular Devices, CA, USA) at 550 nm. The intracellular iron levels were normalized to the protein concentrations determined by the Bradford assay (Bio-Rad, CA, USA).

### Statistical analysis.

A chi-square test was performed when proportions were compared. Student’s *t* test was performed for comparative analyses between two groups. GraphPad Prism (version 8.0.1; GraphPad Software Inc., CA, USA) was used for statistical analysis.

### Data availability.

The GenBank accession numbers for the genome sequences of all 79 C. jejuni isolates used in this study are presented in Table S2 in the supplemental material.
