# Supplementary material for: Phylogenetic Association and Genetic Factors in Cold Stress Tolerance in Campylobacter jejuni
Source: Microbiol Spectr. 2022 Oct 31;10(6):e02681-22. doi: 10.1128/spectrum.02681-22 (PMC9769813; doi:10.1128/spectrum.02681-22)
Supplement: Supplemental file 1 — Supplemental material. Download spectrum.02681-22-s0001.pdf, PDF file, 0.4 MB [file spectrum.02681-22-s0001.pdf]

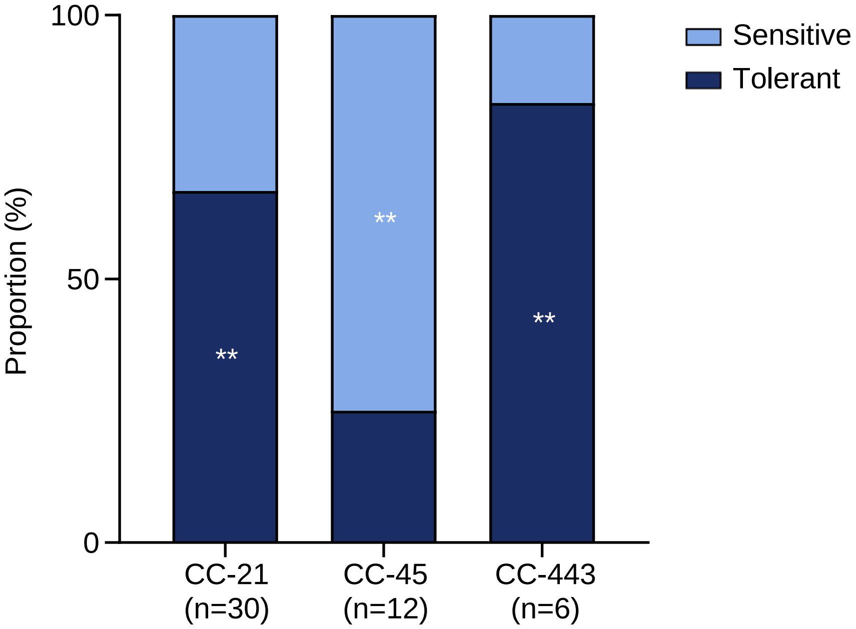

Figure S1. Distribution of cold stress tolerance of strains among CC-21, CC-45, and CC-443. The proportions of cold stress-sensitive and cold stress-tolerant strains among CC-21, CC-45, and CC-443 were compared. A chi-square test was conducted for comparison of the proportions of cold stress-tolerant strains in the CCs. **, $P < 0.01$; CC, clonal complex.

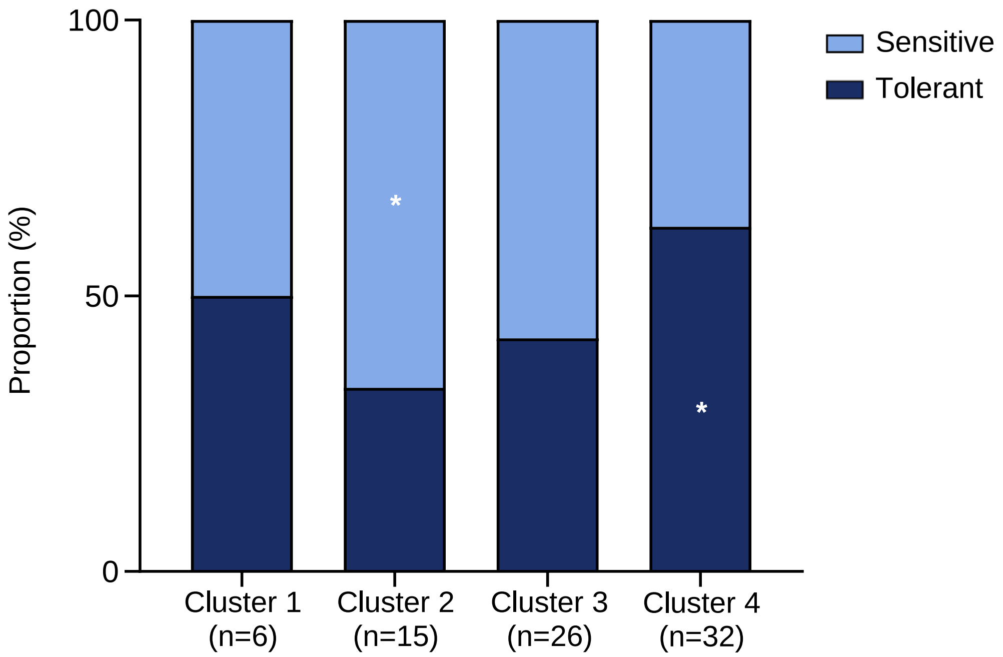

Figure S2. Distribution of cold stress tolerance of strains belonging to the four Clusters. The distribution of cold-sensitive strains (n=40) and cold-tolerant strains (n=39) in Cluster 1, Cluster 2, Cluster 3, and Cluster 4 was compared A chi-square test was conducted to compare the proportions of cold stress-tolerant strains in the clusters. *, $P < 0.05$.

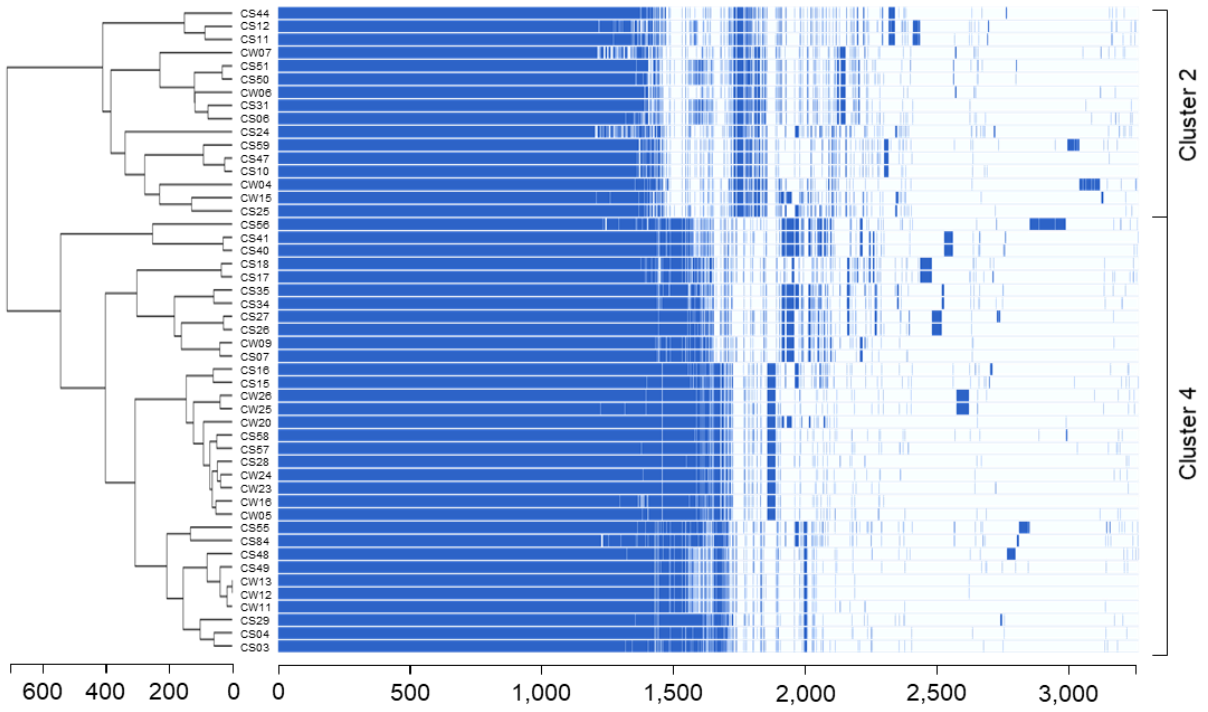

Figure S3. Pan-genome map comparing cold-sensitive cluster (Cluster 2) and cold-tolerant cluster (Cluster 4). A pan-genome map was generated with the 47 strains from Cluster 2 (n=15) and Cluster 4 (n=32).

Table S1. Primers used in this study

Primer	Sequence (5′ to 3′)	Reference
16s-qPCR-F	ATAAGCACCGGCTAACTCCG	1
16s-qPCR-R	TTCCATCTGCCTCTCCCTCA	
kan-F	GCGATGAAGTGCGTAAG	2
kan-R	CGGCTCCGTCGATACTATG	
cfrA-Sal1-F	AAAGTCGACGCTAGCAGTTGAAGATTTGCAAA	This study
cfrA-BamH1-R	AAAGGATCCCTGCGTTTTCGTTTTCAAATTCTTG	
cfrA-inv-F	GGCAAGGTTGAATACAAAGGTGT	
cfrA-inv-R	CCAGTAATACCCCTCATAGTGAT	
cfrA-comp-Not11-F	TTTGCGGCCGCGGTAAGAGCTGGCTTGTGGA	
cfrA-comp-Not1-R	AAAGCGGCCGCGCGTATAGTCGAAGCAGGTAT	
pFMBcomC-inv-Not1-F	GCGGCCGCAAGCTAATTGTTTATGGGGAATTAG	
pFMBcomC-inv-Not1-R	GCGGCCGCTTATTACTTTGTACTCTAGGGGT	
kan-conf-F	CGGGGAAGAACAGTATGTCG	
kan-conf-R	CTCCCACCAGCTTATATACCTTA	
pFMBcomC-F2	CCTGTTTCTATGATACCGTGGA	
pFMBcomC-R3	GGGCCTAACAAGACTTGAACTT	
cfrA-conf-F1	AATGCCTACAAAATCAAAGATAGTGATA	
cfrA-conf-F2	AAAGTCCAGGTAAATTCTACAAGAAC	
cfrA-conf-F3	AAAGGATAATGCACCTATTGGTTCTA	
cfrA-conf-F4	TTACAGGCTTTAGAACCCCTTATG	
cfrA-conf-R1	GTGGGGAGGTTCTTGTAGAATT	
cfrA-conf-R2	TTTTCTAGAGAGCCACTCCATGTTTTTCAAG	
cfrA-conf-R3	ATTCCTGGACTTGTGATGGTTCC	
cfrA-conf-R4	CTGCCTTGGCCACTATAACTG	
cfrA-P/A-F	TTTGTCGCAGAAGATATTATCTTAGATA	
cfrA-P/A-F	TTTGTATCACCCATATAGCGATCTATTT	
cfrA-RT-F	CCTGCTACTATCAATGTTATCAC	
cfrA-RT-R	CTGACGACGCCCATCAATCA	

Table S2. The GenBank accession numbers of the genome sequences of the 79 *C. jejuni* isolates used in the study

Sample name	Genome Accession
CS01	JAMGEB000000000
CS02	JAMGEA000000000
CS03	JAMGDZ000000000
CS04	JAMGDY000000000
CS05	JAMGDX000000000
CS06	JAMHFV000000000
CS07	JAMGDW000000000
CS08	JAMGDV000000000
CS09	JAMGDU000000000
CS10	JAMHFU000000000
CS11	JAMGDT000000000
CS12	JAMGDS000000000
CS13	JAMGDR000000000
CS14	JAMGDQ000000000
CS15	JAMHFT000000000
CS16	JAMHFS000000000
CS17	JAMHFR000000000
CS18	JAMGDP000000000
CS19	JAMHFQ000000000
CS22	JAMGDO000000000
CS23	JAMGDN000000000
CS24	JAMGDM000000000
CS25	JAMGDL000000000
CS26	JAMGDK000000000
CS27	JAMGDJ000000000
CS28	JAMGDI000000000
CS29	JAMHFP000000000
CS31	JAMGDH000000000
CS32	JAMGDG000000000
CS33	JAMGDF000000000
CS34	JAMGDE000000000
CS35	JAMGDD000000000
CS36	JAMGDC000000000
CS37	JAMGDB000000000
CS38	JAMGDA000000000
CS40	JAMGCZ000000000
CS41	JAMGCY000000000

CS42	JAMHFO000000000
CS43	JAMHFN000000000
CS44	JAMHFM000000000
CS45	JAMHFL000000000
CS47	JAMGCX000000000
CS48	JAMGCW000000000
CS49	JAMGCV000000000
CS50	JAMGCU000000000
CS51	JAMHFK000000000
CS52	JAMHFJ000000000
CS53	JAMGCT000000000
CS54	JAMHFI000000000
CS55	JAMWEZ000000000
CS56	JAMGCS000000000
CS57	JAMHFH000000000
CS58	JAMHFG000000000
CS61	JAMGCR000000000
CS62	JAMGCQ000000000
CS63	JAMHFF000000000
CS64	JAMHFE000000000
CW01	JAMGCP000000000
CW02	JAMGCO000000000
CW03	JAMGCN000000000
CW04	JAMGCM000000000
CW06	JAMGCL000000000
CW07	JAMGCK000000000
CW08	JAMGCJ000000000
CW09	JAMGCI000000000
CW11	JAMGCH000000000
CW12	JAMGCG000000000
CW13	JAMGCF000000000
CW15	JAMGCE000000000
CW16	JAMHFD000000000
CW17	JAMGCD000000000
CW18	JAMGCC000000000
CW19	JAMGCB000000000
CW20	JAMGCA000000000
CW22	JAMGBZ000000000
CW23	JAMGBY000000000
CW24	JAMGBX000000000
CW25	JAMGBW000000000

CW26	JAMGBV000000000

References

1. Kim J, Hur JI, Ryu S, Jeon B. 2021. Bacteriophage-mediated modulation of bacterial competition during selective enrichment of *Campylobacter*. Microbiol Spectr 9:e0170321. https://doi.org/10.1128/spectrum.01703-21.
2. Oh E, Jeon B. 2014. Role of Alkyl Hydroperoxide Reductase (AhpC) in the Biofilm Formation of *Campylobacter jejuni*. PloS One 9:e87312. https://doi.org/10.1371/journal.pone.0087312.
